# Characterization of Bacterial Microbiota of P.D.O. Feta Cheese by 16S Metagenomic Analysis

**DOI:** 10.3390/microorganisms9112377

**Published:** 2021-11-17

**Authors:** Panagiotis Papadakis, Spyros Konteles, Anthimia Batrinou, Sotiris Ouzounis, Theofania Tsironi, Panagiotis Halvatsiotis, Efstathia Tsakali, Jan F. M. Van Impe, Despina Vougiouklaki, Irini F. Strati, Dimitra Houhoula

**Affiliations:** 1 Department of Food Science and Technology, University of West Attica, 28 Agiou Spiridonos Str., 12243 Egaleo, Greece; ppapadakis23@gmail.com (P.P.); skonteles@uniwa.gr (S.K.); batrinou@uniwa.gr (A.B.); dvougiouklaki@uniwa.gr (D.V.); estrati@uniwa.gr (I.F.S.); 2 Department of Biomedical Engineering, University of West Attica, 28 Agiou Spiridonos Str., 12243 Egaleo, Greece; souzounis@uniwa.gr; 3 Department of Food Science and Human Nutrition, Agricultural University of Athens, 75 Iera Odos, 11855 Athens, Greece; ftsironi@aua.gr; 4 2nd Propaedeutic Department of Internal Medicine, Medical School, National and Kapodistrian University of Athens, “ATTIKON” University Hospital, 1 Rimini Str., 12462 Chaidari, Greece; pahalv@gmail.com; 5 Department of Chemical Engineering, BioTeC+—Chemical and Biochemical Process Technology and Control, KU Leuven, Gebroeders De Smetstraat 1, 9000 Gent, Belgium; jan.vanimpe@kuleuven.be

**Keywords:** feta cheese, 16S metagenomic analysis, microbiota biodiversity

## Abstract

Background: The identification of bacterial species in fermented PDO (protected designation of origin) cheese is important since they contribute significantly to the final organoleptic properties, the ripening process, the shelf life, the safety and the overall quality of cheese. Methods: Ten commercial PDO feta cheeses from two geographic regions of Greece, Epirus and Thessaly, were analyzed by 16S metagenomic analysis. Results: The biodiversity of all the tested feta cheese samples consisted of five phyla, 17 families, 38 genera and 59 bacterial species. The dominant phylum identified was Firmicutes (49% of the species), followed by Proteobacteria (39% of the species), Bacteroidetes (7% of the species), Actinobacteria (4% of the species) and Tenericutes (1% of the species). Streptococcaceae and Lactobacillaceae were the most abundant families, in which starter cultures of lactic acid bacteria (LAB) belonged, but also 21 nonstarter lactic acid bacteria (NSLAB) were identified. Both geographical areas showed a distinctive microbiota fingerprint, which was ultimately overlapped by the application of starter cultures. In the rare biosphere of the feta cheese, *Zobellella taiwanensis* and *Vibrio diazotrophicus*, two Gram-negative bacteria which were not previously reported in dairy samples, were identified. Conclusions: The application of high-throughput DNA sequencing may provide a detailed microbial profile of commercial feta cheese produced with pasteurized milk.

## 1. Introduction

Feta cheese is a salty and slightly acidic traditional white soft cheese of ancient Greek origin characterized as PDO (protected designation of origin) by the European Community which accounts for approximately 10% of the Greek food exports due to its significant international reputation [1]. PDO feta cheese is only produced with the milk from native breeds of ewes and goats in the geographic areas of Macedonia, Western Thrace, Epirus, Thessaly, Central Greece, Peloponnese, and Lesvos Island. Traditionally, feta cheese may be produced from raw milk obtained from ovine milk or its mixture with caprine milk (up to 30%) at small-scale farms and rennet from lambs and young goats, but in industrial large-scale production, pasteurized milk is used [2] while LAB starter cultures are used for the initial acidification of the milk along with calcium chloride (2% max) and commercial rennet. The most commonly used starter cultures are *Lactococcus lactis*, *Lactobacillus delbrueckii* subsp. *bulgaricus*, *Streptococcus thermophilus*, *Leuconostoc lactis* [3,4]. However, like in any fermented food complex, the microbial communities that colonize the milk used for feta production play a significant role in forming the rich flavor and aroma of the final product [2,5,6]; for example, *Enterococcus faecium* may also be an important part of the microbiota of other traditional cheeses, although some strains may harbor genetic determinants of virulence [7]. The salting of the curd assists with the development of microbiota, and maturation is a two-stage process after the curd is placed in containers and filled up with 7% (*w*/*w*) brine, lasting for a minimum of two months. Recent studies exploring the microbiota of artisanal cheese with non-culturable methods have revealed highly heterogenous microbial ecosystems that could not be detected with the standard microbiological methods [5,8]. It is important to characterize the biodiversity of feta cheese at the species level since these various microorganisms can play vital roles in the development of the organoleptic properties of cheese, nutrient composition, ripening, taste, aroma, shelf life and safety [9,10]. Moreover, the microbial fingerprint of feta cheese may provide details of the hygienic conditions that exist throughout all the stages of production at various small- or large-scale facilities. The application of high-throughput DNA sequencing, such as NGS, can facilitate the detection of all the microbial species that were present from farm to plant and up to the moment of the specific analysis, providing an extensive picture of the microbial “history” of the cheese by identifying various categories of microorganisms of the microbial community. This thorough analysis can reveal (a) active species found in abundant quantities, (b) inactive species that may be either dead or metabolically lethargic due to environmental stresses, (c) adventitious species, (e) subdominant populations that could be overlooked if the samples were analyzed by means of culture-dependent methods, (f) species belonging to the rare “biosphere” found in very low abundance and, (g) in some cases, species that were identified for the first time in specific food ecosystems [11]. The aim of this study was to explore by 16S metagenomic analysis the bacterial microbiota of various commercial Greek PDO feta cheeses in order to characterize the species that form the bacterial communities of the products and compare the microbial profiles of feta cheese produced in two specific geographic regions, Epirus and Thessaly, that are major feta producers.

## 2. Materials and Methods

### 2.1. Preparation of Samples

A total of 10 feta cheese samples in a prepacked format from ten different manufacturers and two regions (five manufacturers from each region, Epirus and Thessaly) were obtained from supermarkets in Athens, Greece, and transported to the Department of Food Science and Technology (University of West Attica, Egaleo, Greece). The samples were transported directly to the laboratory and kept at −18 °C until analyzed. The samples were selected so that they all have similar expiration dates.

### 2.2. DNA Extraction and Next-Generation Sequencing

The cheese samples were aseptically homogenized in a laminar flow hood. DNA extraction was performed using a NucleoSpin Food kit (Macherey-Nagel, GmbH & Co. KG, Düren, Germany) according to the manufacturer’s instructions with some modifications. Approximately 100 mg of each sample were used for the extraction after grinding in liquid nitrogen. The sample was incubated with the lysis buffer and Proteinase-K overnight at 65 °C. After the lysis, the precipitation with absolute ethanol and the washing steps, DNA was eluted in duplicate in order to increase the concentration. The purity and the quantity of the extracted DNA was evaluated spectrophotometrically by calculating OD260/OD280 (spectrophotometer Epoch, BioTek, Winooski, VT, USA).

After DNA extraction, 16S rRNA genes were amplified using domain-level bacterial primers that contain sequencing adapters and unique sample-specific sequences. The 16S subunit of ribosomal RNA (16S rRNA) is used to classify bacteria. Seven different 16S rRNA hypervariable regions (V2, V3, V4, V6–7, V8 and V9) in each sample were amplified. The 16S subunit of rRNA consists of approximately 1500 nucleotide bases and is found in all bacteria. Random changes are an indicator of the evolution of bacteria. Despite the high homology of 16S rRNA among bacterial species, it contains in its sequence nine variable regions that are distinct between the different microbes and which can therefore be used for identification and discrimination between bacteria.

Multiple samples barcoded and sequenced simultaneously on a single Ion PGM 318 chip resulted in a sufficient number of reads. The number of total reads per sample was between 2.5 × 10^6^ and 4.5 × 10^6^. Approximately 50–65% of these reads passed stringency filters, and of these, 60–75% mapped to the databases. The primers included in the kit were used to amplify 16S variable regions from the samples. After generating amplicons, an Ion Plus™ Fragment Library Kit was used to ligate barcoded adapters and synthesize libraries. Barcoded libraries from all the 10 samples were pooled and templated on a OneTouch2™ system followed by 400 bp sequencing on the Ion PGM. Automated analysis, annotation and taxonomic assignment occurred via the Ion Reporter Software pipeline. Classification of reads was through alignment to either the curated MicroSEQ ID or curated Greengenes databases.

### 2.3. Bioinformatic and Statistical Analyses

In order to explore and identify the key species in the feta microbiota, a series of statistical tests were applied. The analyses were carried out both in the whole microbiome and in the microbiome after the three major starter species removal (*Streptococcus thermophilus*, *Lactobacillus delbrueckii* and *Lactococcus lactis*). At first, the number of species in each sample from Thessaly and Epirus was calculated. Then, the Kruskal–Wallis test was used to investigate whether the variance of the species numbers differs significantly between the two regions [12]. Moreover, in order to quantify the diversity of each region, the Shannon diversity index was employed, which measures the alpha diversity [13,14] of each region by considering species abundance and evenness. At this point, the Kruskal–Wallis test was applied once again to investigate whether the diversity of the Thessaly and Epirus microbiota differed statistically. Those analyses were performed using the R programming language and the vegan package [15]. Furthermore, beta diversity [13] was used as a measure of dissimilarity between the two regions. More specifically, the within-sample divergence was calculated in order to quantify the overall heterogeneity in community composition across samples. So, as beta diversity, the average dissimilarity of each sample from the group median was considered. Moreover, the beta diversity of the two regions was investigated to determine whether they differed significantly. Those calculations were performed with the help of the microbiome package [16]. The next step of communities’ exploration was to check whether the microbiome composition was affected by the region of sample collection. Nonparametric permutational multivariate analysis of variance (perMANOVA) [17] was implemented with the function adonis2, based on the Bray–Curtis dissimilarity matrix [15]. This test revealed the differences in microbiome composition. The null hypothesis of the perMANOVA test was that the two regions do not differ in spread or position in the multivariate space. Principal component analysis (PCA) [18] was also applied to the species OTU counts to have a visual inspection of the data distribution and identify any clusters among the samples. For the analysis and results visualization, the vegan and ggplot2 packages were used [19]. In addition, to further explore the clustering that occurs among the data, the species abundances for each sample was calculated. Then, a heatmap was constructed by applying the k-means clustering algorithm [20] in order to cluster the data according to their abundance values. The k (number of clusters) was set to two since there were two clusters in this particular study (two different geographic regions). The heatmap was designed using the package pheatmap [21]. Analysis of the core microbiota was also implemented by identifying the species relative population frequencies at 1% compositional abundance and 75% prevalence for each region. For this analysis, the microbiome and eulerr packages were used [22]. The core microbiota identification was carried out with the microbiome package. In addition, the exploration of clustering among the samples based on species dissimilarity was performed by means of a hierarchical clustering [23] according to Bray–Curtis dissimilarity. Bray–Curtis dissimilarity is zero for samples that have the same composition and one for those that have completely different species. For this analysis, the package dendextend was used [24]. Then, we applied Ward’s clustering algorithm [25], which finds the pair of clusters at each iteration that minimizes the increase in total variance. Finally, the Wilcoxon test was performed for each species so as to identify which of them had statistically different OTU counts between the two regions [26].

## 3. Results

### 3.1. Bacterial Diversity of Feta Cheese

**Figure 1:**
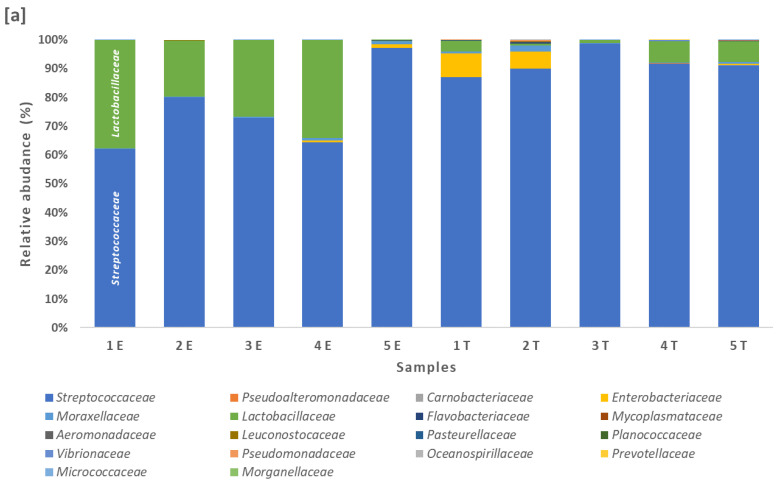

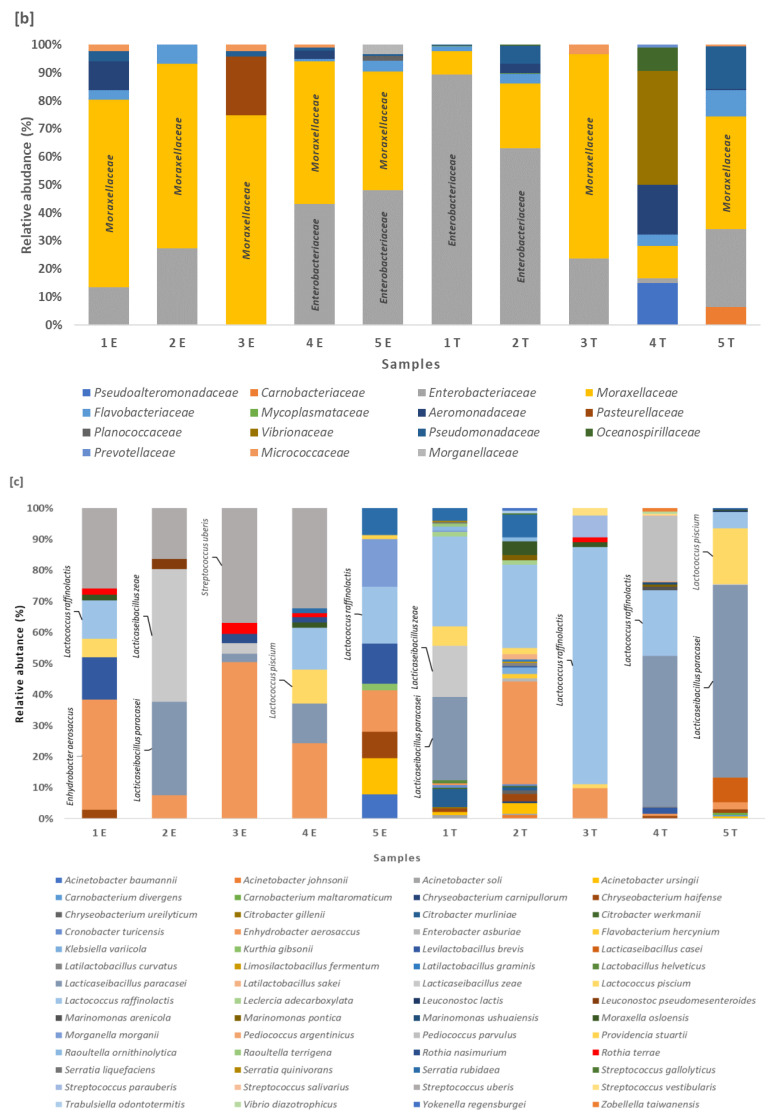
Bar plots of relative abundance (%) (**a**) of bacterial families across the sampling regions (E: Epirus; T: Thessaly). Bacterial families that did not reach the minimum of 0.01% were omitted; the family *Streptococcaceae* prevailed in all the samples, mainly due to the presence of the starter culture *Streptococcus thermophilus*, although there were obvious variations in percentages among the samples; (**b**) of bacterial families across the sampling regions after the exclusion of the families *Streptococcaceae* and *Lactobacillaceae*, to which the three main starter species belong; the background subdominant microbiota emerged, and especially the families Enterobacteriaceae and *Moraxellaceae* that have characteristic Gram-negative bacteria present in most food products of animal origin; (**c**) of bacterial species in all the feta cheese samples excluding the three starter LAB species *Streptococcus thermophilus*, *Lactobacillus delbrueckii* and *Lactococcus lactis*. The coding in the *x*-axis refers to the five samples from Epirus (1E–5E) and the five samples from Thessaly (1T–5T).

The biodiversity of all the feta cheese samples analyzed consisted of 59 bacterial species corresponding taxonomically to 38 genera, 17 families and five phyla. The dominant phylum identified was Firmicutes (49% of the species), followed by Proteobacteria (39% of the species), Bacteroidetes (7% of the species), Actinobacteria (4% of the species) and Tenericutes (1% of the species). The Gram-positive species belonging to the phylum of Firmicutes, especially lactococci, lactobacilli and streptococci, were dominant in the microbiota of all the samples (Figure 1a). These results are consistent with the findings of similar studies that have revealed the prevalence of Firmicutes and, in particular, of the *Lactococcus*, *Lactobacillus* and *Streptococcus* genera in goat and sheep milk and cheese [27,28,29,30,31]. The second most abundant phylum was Proteobacteria, which is one of the most diverse phyla of Gram-negative bacteria with many known human pathogens and in which the various species of *Enterobacteriaceae* prevailed, followed by Moraxellaceae (Figure 1b).

#### 3.1.1. Firmicutes: The Dominant Phylum

In Firmicutes, four families of Gram-positive bacteria were detected with a concentration > 0.01% in at least one of the cheese samples, with *Streptococcaceae* and *Lactobacillaceae* being the most dominant, followed by *Carnobacteriaceae* and *Planococcaceae*. The lactic acid bacteria (LAB) were represented by ten genera: *Lactobacillus*, *Levilactobacillus, Lacticaseibacillus*, *Latilactobacillus*, *Limosilactobacillus*, *Carnobacterium*, *Lactococcus*, *Leuconostoc*, *Pediococcus* and *Streptococcus*. Twenty-four LAB species were present in at least one of the samples, including the three main starter cultures of *Streptococcus thermophilus*, *Lactobacillus delbrueckii* and *Lactococcus lactis*, and overall 21 nonstarter LAB species were detected (Table 1). Figure 1c shows the bacterial species that were detected in the feta cheese samples with a > 0.01% abundance, excluding the three main starter LAB species that were found in very large percentages in all the samples.

#### 3.1.2. Gram-Negative Bacteria

The Gram-negative bacteria that were identified in the feta cheese studied consisted of a highly heterogenous population represented by 26 genera belonging to two phyla, Proteobacteria and Bacteroidetes. In the phylum of Proteobacteria, which is one of the most abundant phyla of the gut microbiota, ten families of Gram-negative bacteria were identified with a concentration > 0.01% in at least one of the cheese samples. The most abundant was the family of *Enterobacteriaceae*, which was dominant in all the samples and consisted of nine genera, six of which are considered coliforms: *Citrobacter* (four species), *Enterobacter* (one species), *Klebsiella* (one species), *Serratia* (three species), *Raoultella* (two species) and *Leclercia* (one species).

### 3.2. Bioinformatic and Statistical Analysis

**Figure 2:**
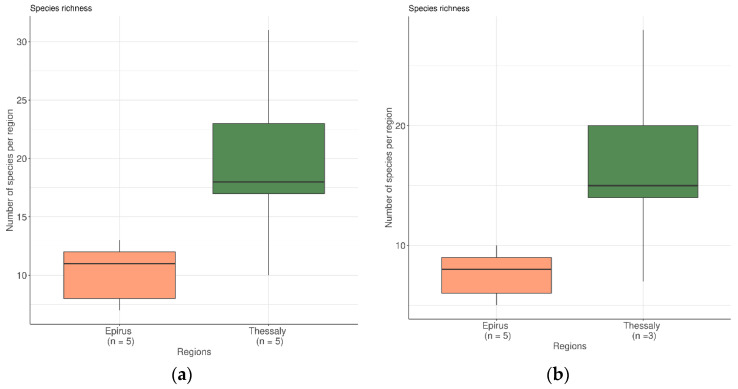
(**a**) Bar plot of the species richness for the two regions; all the species were included in the analysis (*p* = 0.0472); (**b**) Bar plot of the species richness for the two regions after the three major species were removed from the analysis (*p* = 0.0472).

In order to investigate the microbial ecology of the feta cheese samples, various indices of α-diversity (the mean diversity of species) were calculated to assess the differences between microbial environments. These indices included the species richness which is the number of species in each sample and the Shannon diversity index (Si), which considers the number of species living in a habitat and their relative abundance. A large Shannon diversity index indicates the presence of many species with balanced abundances. The difference in variance of species richness between the sampling regions (Epirus and Thessaly) was tested using the Kruskal–Wallis test. Initially, the test was carried out including all the species identified by NGS. The test showed that species richness between the two regions differed significantly (*p* = 0.0472). More specifically, within the five Epirus samples, 11 species were included, whereas the samples from Thessaly had 18 species (Figure 2a). Then, in the second analysis, the species that are usually used as starter cultures and thus populationally prevail (*Lactobacillus delbrueckii*, *Lactococcus lactis*, *Streptococcus thermophilus*) were excluded. The species richness was also significantly different between the two regions (*p* = 0.0472) (Figure 2b).

**Figure 3:**
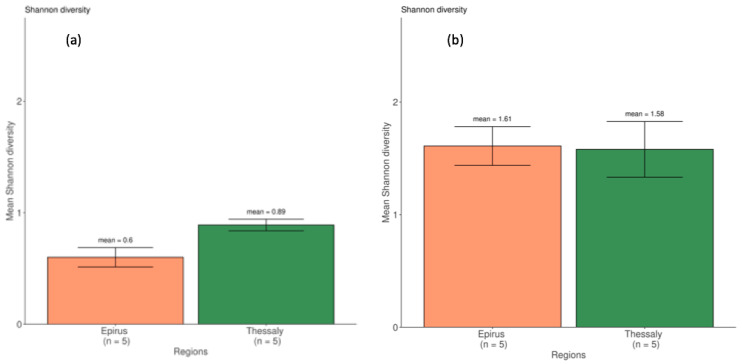
(**a**) Bar plot of the Shannon diversity between the two regions; all the species were included in the analysis, *p* = 0.0282; (**b**) Bar plot of the Shannon diversity between the two regions after the three major species were removed from the analysis, *p* = 0.916.

The difference in the Shannon diversity index, using the Kruskal-Wallis test, was calculated for each sample per region, and it was found that the two regions significantly differed (Figure 3a) in species diversity, *p* = 0.0282 (all the species included in the analysis). In particular, the five Epirus samples had a mean Shannon index = 0.6, while the five samples from Thessaly had a mean Si = 0.89 (Figure 3a). The same analysis was carried without the three starter cultures species. In that case, it was found that the Shannon diversity index of the two regions did not differ (*p* = 0.916). The five Epirus samples had a mean Si = 1.61 and the five samples from Thessaly had a mean Si = 1.58 (Figure 3b). Practically, these results show that the starter culture combinations are not standardized amongst the different samples and regions.

**Figure 4:**
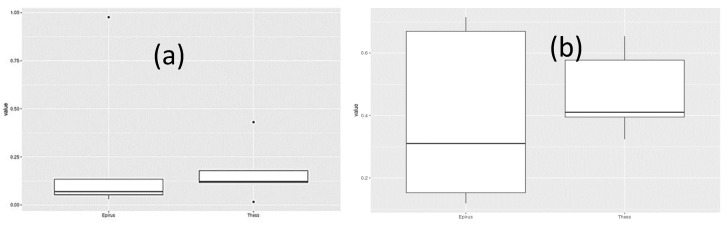
Boxplots of the divergences within the Epirus and Thessaly groups with respect to the median profile within each group. (**a**) all the species included in the analysis (*p* = 0.841); (**b**) after the three major species were removed from the analysis (*p* = 0.151).

In addition, beta diversity was calculated, which quantified dissimilarity in community composition between the Epirus and Thessaly samples. More specifically, divergence was used in order to quantify the dissimilarity. Divergence within a sample set quantifies the overall heterogeneity in community composition across samples. In this study, beta diversity was quantified as the average dissimilarity of each sample from the group median, and then the median between regions was evaluated using the Wilcoxon test in order to evaluate whether the beta diversity between the two regions differs significantly. The test yielded *p* = 0.841 indicating that there was no statistical significance (all the species included in the analysis) in terms of the heterogeneity of the regions (Figure 4). The same test was performed after the three major species were removed from the analysis, and it yielded *p* = 0.151, indicating that there was no statistical significance in terms of the heterogeneity of the regions.

Moreover, nonparametric permutational multivariate analysis of variance (perMANOVA) with the function adonis2, based on the Bray–Curtis dissimilarity matrix, was performed. The analysis included all the species and yielded *p* = 0.054, which means that there is no statistically significant effect of the region on the microbiome composition, whereas when the three major species were removed from the analysis, perMANOVA yielded *p* = 0.016, which means that there is a strong statistically significant effect of the region on the microbiome composition.

**Figure 5:**
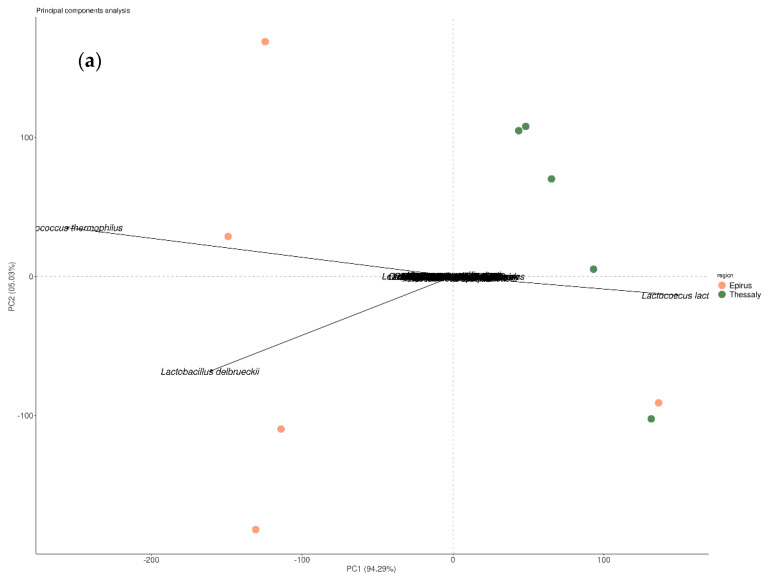

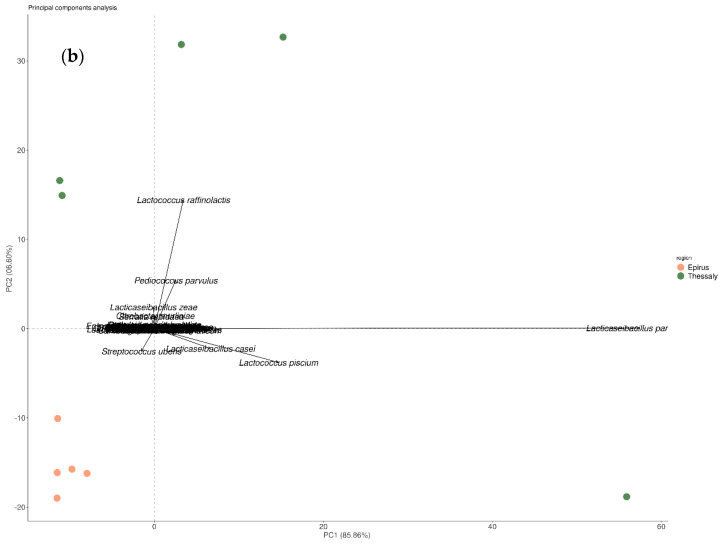
Principal component analysis (PCA) plot of the two first principal components. (**a**) PC1 explains 94.29% of the data variance and PC2 explains 5.03% of the variance when all the species are included in the analysis; (**b**) PC1 explains 85.86% of the data variance and PC2 explains 6.60% of the variance after the three major species are removed from the analysis.

Principal component analysis (PCA) was carried out in order to investigate clustering of the species of the samples (Figure 5). The points in the PCA plot indicate the samples and arrows are the species. The length of the arrow indicates the amount of variation in the studied communities explained by that particular variable (longer arrows meaning larger variation) and the angle of the arrows to each other indicates correlations (the more obtuse the angle, the less correlated). In Figure 5b, PCA shows clustering of the Epirus samples after the three main starter species were excluded from the analysis.

**Figure 6:**
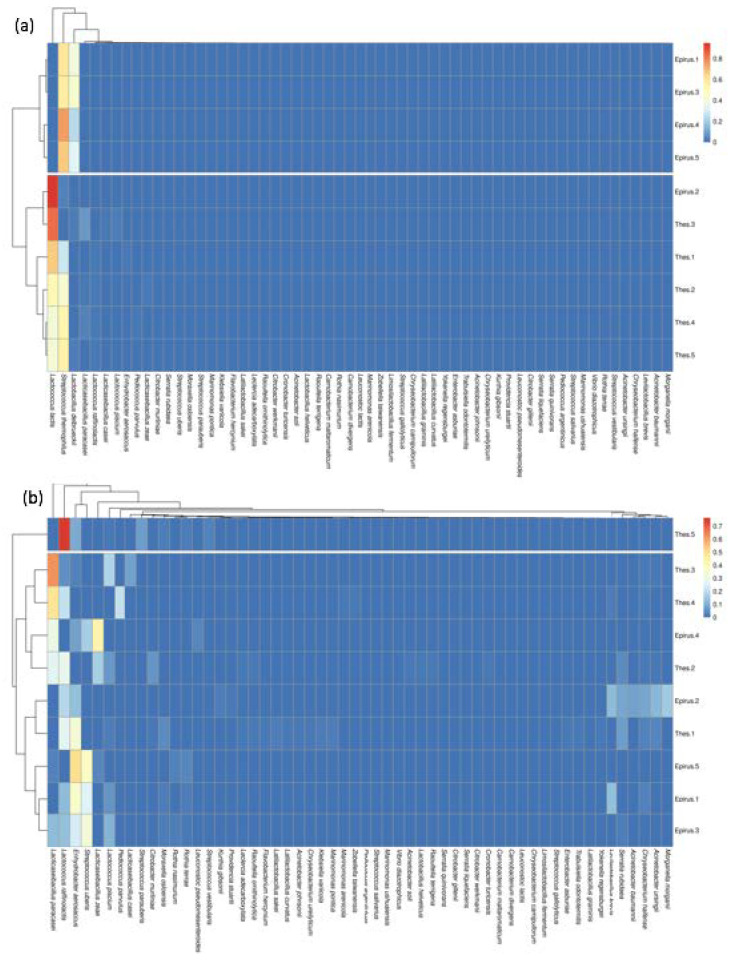
Heatmap of the abundance values that each sample has among the species. The cells of the heatmap are colored according to the abundance. (**a**) The graph produced with all the species in the analysis; (**b**) The graph produced after the three major species were removed from the analysis.

The heatmap in Figure 6 is designed by applying the k-means clustering algorithm in order to cluster the data according to their values. The k (number of clusters) was set to 2 (two different regions) in order to investigate whether the samples could be classified correctly according to their abundance values. The *x*-axis represents the species and the *y*-axis represents the samples.

**Figure 7:**
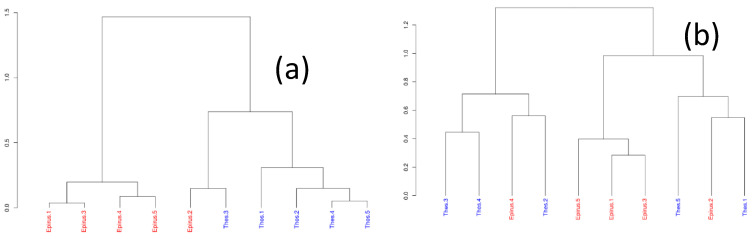
Dendrogram produced from Ward’s clustering according to Bray–Curtis dissimilarity (**a**) with all the species included; (**b**) after the three major species were removed.

In Figure 6a, the two clusters are separated from the white horizontal stripes, while the dendrograms reflect which species/samples belong to the same cluster. As can be seen, the first cluster contains four out of the five samples from Epirus, and in the second cluster, all the samples from Thessaly are grouped plus one sample from Epirus. In Figure 6b, after the removal of the three major species, the samples are clustered in a completely different way. The first cluster contains only one sample from Thessaly, whereas all the other samples from Thessaly and Epirus are clustered together. However, in the dendrogram on the left side of the Figure 7, it can be observed that the second cluster consists of two major subclusters. One subcluster contains three out of the five Thessaly samples, whereas the second subcluster contains four out of the five Epirus samples. 

Analysis of the core microbiota was implemented by identifying the species relative population frequencies at 1% compositional abundance and 75% prevalence for each region. Then, a Venn diagram was designed in order to identify the common core microbiomes between the regions. According to the Venn diagram of all the species, in the five samples from Thessaly, four species were identified as the core microbiota. Similarly, the five samples from Epirus had four species as the core microbiota. The two regions have only two common species identified in the core microbiome, *Lactobacillus delbrueckii* and *Streptococcus thermophilus*, which are the starter cultures. The other two species found in the core microbiome of Epirus were *Enhydrobacter aerosaccus* and *Streptococcus uberis*. The other two species found in the core microbiome of the Thessaly samples were *Lactococcus raffinolactis* and *Lactococcus lactis*.

In order to have a better understanding of the clustering that occurs among the samples based on species dissimilarity, hierarchical clustering according to Bray–Curtis dissimilarity was also performed (Figure 7). Bray–Curtis dissimilarity is zero for samples that have the same composition and one for those that have completely different species. Ward’s clustering algorithm was applied, which finds the pair of clusters at each iteration that minimizes the increase in total variance. In Figure 7a, when all the species were included in the analyses, four out of the five Epirus samples were clustered together according to their Bray–Curtis dissimilarity values. This indicates that the species composition of those samples is different from that of the Thessaly samples. However, in the Thessaly samples cluster, we detected one sample from the Epirus region, indicating that this sample has similar species composition with the Thessaly samples. In Figure 7b, when the three major species were excluded from the analyses, four out of the five Epirus samples were clustered together according to their Bray–Curtis dissimilarity values. However, in this major cluster, two other samples from Thessaly were also found. This indicates that the species composition of those samples is similar. In the other major cluster, three Thessaly samples and one Epirus sample were detected. Moreover, the subclusters created by Ward’s algorithm show that there were mixtures of the Thessaly and Epirus samples clustered together. This is an indication that if the three major species are removed, the samples cannot be clustered clearly according to their region.

## 4. Discussion

All the feta cheese samples were produced with pasteurized milk and commercial LAB starters; therefore, the three specific species commonly used as starters *Streptococcus thermophilus*, *Lactobacillus delbrueckii* and *Lactococcus lactis* were observed with the highest abundance, although in different proportions in each sample. However, the presence of high diversity of nonstarter LAB species (NSLAB) in commercial PDO cheese is also significant since they form part of the natural microbiota of milk as well as the equipment and processing environment and may contribute significantly to the maturation and the development of organoleptic characteristics of the cheese. These results are in agreement with other studies that have assessed the microbiota in raw milk feta cheese [29] and in raw milk artisanal dairy products [32] with molecular methods and have revealed a high degree of genetic polymorphism among the NSLAB at the strain and species levels. The presence of 21 NSLAB species in low percentages in the feta cheese samples (Table 1) detected in this study underlines the significance of extended LAB diversity even in pasteurized milk that may be linked to aromatic, flavor and texture variations of the final cheese product. These results also indicate that culture-independent molecular methods can identify a more diverse bacterial population in feta cheese than previously estimated. Many of these bacteria produce various organic compounds and extracellular enzymes that retain their biological activity even after pasteurization [33]. Moreover, it is probable that some of the non-thermotolerant bacteria within these populations may survive pasteurization and are likely to be in the nonculturable form and cannot be detected with the standard culture methods [34].

In the family of *Lactobacillaceae*, 13 species were identified corresponding to seven genera, of which four were former *Lactobacillus* which were recently reclassified to distinct new genera [35]. Among the most common species observed was *Lacticaseibacillus paracasei* (former *Lactobacillus paracasei*), a facultative heterofermentative LAB detected in six out of the ten samples. *L. paracasei* can be commonly found as the dominant nonstarter lactic acid bacterium in many ripened cheeses, and select strains of this species may be added to cheese milk as adjuncts for outcompeting undesired microorganisms and improving cheese quality by contributing to cheese flavor diversification and intensification [36]. Moreover, the probiotic properties of *L. paracasei* have been investigated in recent studies [37]. Other facultative heterofermentative NSLAB detected in very low populations were *Lacticaseibacillus casei* (former *Lactobacillus casei*), *Latilactobacillus graminis* (former *Lactobacillus graminis*), *Latilactobacillus sakei* (former *Lactobacillus sakei*) and *Lacticaseibacillus zeae* (former *Lactobacillus zeae*).

*Levilactobacillus brevis* (former *Lactobacillus brevis*), mesophilic or psychrotrophic lactobacilli, were found in four samples. *L. brevis* is heterofermentative, using the phosphoketolase pathway to produce a mixture of lactic acid, ethanol, acetic acid and CO_2_ as the products of hexose fermentation [38]. *L. brevis* and *Limosilactobacillus fermentum* (former *Lactobacillus fermentum*, also heterofermentative bacilli) are the most common adventitious NSLAB found in cheese produced with non-pasteurized milk; however, in most cases, strains enter cheese after pasteurization, e.g., during brining [39]. These two species have also been identified in feta cheese, and *L. brevis*—in artisanal cheeses produced on the Island of Naxos, Greece, from raw sheep and goat milk [9,40]. Moreover, these gas-forming lactobacilli can cause structural defects and spoil cheeses [39]. *Latilactobacillus curvatus* (former *Lactobacillus curvatus*), also heterofermentative lactobacilli, were detected in two of the samples. In the family of *Streptococcaceae*, *Lactococcus raffinolactis*, which were observed in all the samples, are mesophilic lactic acid bacteria present in a wide range of environments, such as foods (meat, fish, various raw milks, cheese, vegetable) [41]. In the family of *Carnobacteriaceae*, two species were identified, *Carnobacterium divergens* and *Carnobacterium maltaromaticum*, which are found in animals or products of animal origin including dairy, fish and meat and are considered psychrotrophic spoilage LAB that commonly contaminate food processing environments [42].

Metagenomic analysis of the commercial feta samples also revealed the presence of a subdominant community of Gram-negative bacteria in which Enterobacteriaceae were the most abundant in all the samples. To the family of Enterobacteriaceae belong the coliforms which are aerobic or facultative anaerobic Gram-negative rods capable of fermenting lactose by producing gas and acid; they also possess β-d-galactosidase activity. Coliform bacteria are commonly used as microbiological indicators of poor hygienic conditions and therefore it is important to assess their presence in the dairy industry [43]. They most commonly contaminate milk prior to pasteurization from the environment or animal feces, but post-pasteurization contamination from the processing environment is also observed in many cases [44]. Moreover, it has been observed that a low level of coliforms surviving heat treatment may result in high levels at the end of a production day at cheese-making facilities [45]. 

Of particular concern are psychrotolerant coliforms which are adapted to grow at the refrigeration temperatures used to store milk before pasteurization [33]. The growth of psychrotrophic coliforms in refrigerated milk is usually linked with the production of lipolytic and proteolytic enzymes that can result in physical degradation and unacceptable sensory characteristics of the product [46]. In the study of Masiello et al., 2016, all coliform isolates from commercial pasteurized milk and especially *Citrobacter*, *Enterobacter*, *Hafnia*, *Raoultella* and *Serratia* displayed psychrotolerance at 6 °C by growing at least 2 log over 10 days, and a large percentage of the coliform isolates displayed lipolytic and proteolytic activity. Another significant aspect of the presence of a variety of coliforms in the milk and cheese microbiota is the increasing emergence of multidrug-resistant bacterial strains isolated form dairy products [47]. Moreover, many members of these genera have the ability to form biofilms, thus rendering the bacteria more resistant to stress factors and becoming more persistent sources of contamination and food spoilage [48].

The genus *Citrobacter* consisted of *C. murliniae*, *C. werkmanii* and *C. turicensis*, which were detected in two of the samples, and of *C. gillenii* in one of these two samples. *Citrobacter* is considered an opportunistic pathogen, although it is rarely the primary source of human illness [49]. The genus *Enterobacter* has been identified in raw and pasteurized milk [50]. In this study, only one species of the genus *Enterobacter* was detected, *Enterobacter asburiae*, which is an opportunistic Gram-negative pathogen that has been isolated from milk in several studies [28]. Additionally, only one species of *Klebsiella* was detected, *K. variicola*, which was detected in the same sample as *Enterobacter asburiae*. *K. variicola* is a mastitis pathogen, and is also considered an emerging pathogen in humans with health concerns regarding the development of multidrug resistance genes in the bacterium [51]. The species of the genus *Serratia* can be opportunistic pathogens and were also found to exhibit strong lipolytic and proteolytic activity in milk [33]. *Serratia rubidaea*, which was the most common species detected in five samples at a concentration ranging from 0.01–0.12%, is also able to produce a red pigment named prodigiosin [52]. The other two species were *Serratia liquefaciens* and *Serratia quinivorans* found in one sample. *Raoultella* species can be commonly found in the farm environment and are phenotypically very close to *Klebsiella*. *R. ornithinolytica*, detected in two of the samples, is known to inhabit aquatic environments and has pathogenic potential [53], as well as *R. terrigena* which was detected in one of the samples. *Leclercia* isolates are distributed widely in nature and have been isolated from various environmental sources such as food and water and also from various clinical specimens. The only species identified in two of the samples was *Leclercia adecarboxylata* which has been considered a relatively new and unfamiliar marine pathogen and is a member of the normal gut flora in animals [54].

The other three genera of Enterobacteriaceae which were part of the subdominant population of the feta cheese samples were *Cronobacter, Yokenella* and *Trabulsiella*. *Cronobacter turicensis* was detected in the same two samples as *Klebsiella variicola* and *Enterobacter asburiae* and is considered an emerging food-borne pathogen which is found in dairy products and has been reported to cause bacteremia and enteritis in immunocompromised people, especially in infants [55]. *Yokenella regensburgei*, a close phylogenetic relative of the coliforms *Citrobacter* and *Klebsiella*, is also considered an opportunistic pathogen [56]. Finally, *Trabulsiella odontotermitis*, also detected in the same sample as *Cronobacter*, was isolated for the first time from the gut of a termite and is also capable of producing cellulases [57]. There are very limited reports of the genus *Trabulsiella* in the dairy industry, one of them being a study in which the bacterium was detected with molecular methods in Pico cheese, an artisanal Azorean food [58]. *Yokenella* and *Trabulsiella* are considered previously overlooked species in the cheese microbiota [8].

To the “rare biosphere” of the feta microbial community belonged another uncommon bacterium, *Zobellella taiwanensis*, which was detected in one of the feta samples. *Zobellella taiwanensis* belongs to the family *Aeromonadaceae* and is a marine Gram-negative facultatively anaerobic bacterium capable of heterotrophic nitrification and aerobic denitrification, identified for the first time in 2006 [59]. No reports were found in the recent literature mentioning the presence of this bacterium in dairy products. Moreover, *Vibrio diazotrophicus*, a Gram-negative nitrogen-fixing bacterium belonging to the family Vibrionaceae, was detected in the same sample as *Zobellella taiwanensis* and has also not been reported in dairy samples. *Vibrio diazotrophicus* is normally found in marine environments and contains several virulence and antibiotic resistance genes [60]. It is probable that these halotolerant bacteria were contaminants of the feta cheese brine, coming from the salt. The significance of the presence of these bacteria in the cheese ecosystem is not clear; however, it is a proof that the application of the 16S metagenomics approach has the potential to detect the presence of these and other previously overlooked taxa, and therefore further research concerning the role of these can be conducted. 

The second most abundant family of Proteobacteria was *Moraxellaceae*, with *Acinetobacter* being the most prevalent Gram-negative genus, present in all the samples, ranging from 0.01% to 0.63%. The identified species were *A. baumannii*, *A. johnsonii*, *A. soli* and *A. ursingii*. *Acinetobacter* is a psychrotrophic contaminant that has been found to contaminate raw goat milk [61], and its psychrotrophic isolates from raw milk can be highly lipolytic [50]. *Moraxella osloensis*, which is a zoonotic pathogen that has been detected in goat milk [30], was also detected in five of the samples in very low concentrations.

Furthermore, other subdominant genera belonging to the phylum of Proteobacteria detected were *Pseudomonas* (family *Pseudomonadaceae*) detected in eight out of the ten samples (0.01–0.58%), which are aerobic and psychrotrophic bacteria able to produce extracellular proteolytic and lipolytic enzymes in milk that may contribute to spoilage during refrigeration [62]; *Aeromonas* (family *Aeromonadaceae*) detected in six out of the ten samples (0.05–0.32%), which are aquatic facultative anaerobic bacteria and minor pathogens [63]; *Pseudoalteromonas* (family Pseudoalteromonadaceae) detected in one of the samples (0.12%), which is a halotolerant and psychrotolerant bacterial genus that produces lipolytic and proteolytic enzymes [8].

The Morganellaceae family was represented by two species, *Morganella morganii* and *Providencia stuartii*. *Morganella morganii*, which is a Gram-negative bacterium commonly found in the intestinal tracts of humans and in the environment, was detected in low levels (0.01–0.08%) in two of the samples. *M. morganii* has evolved as an emergent zoonotic pathogenic bacterium and has been identified in the milk and feces of infected cattle and goats [64]. The strain isolated in the study of Li et al., 2018, was resistant to the antibiotics commonly used in veterinary medicine, and it was concluded that milk contaminated with *M. morganii* should be considered a potential risk for transmission of infection to humans, specifically to immunocompromised hosts [64]. *Providencia stuartii*, an opportunistic pathogen commonly found in animals, in the environment and in foods was also detected in very low levels in one of the samples.

Two more families of Proteobacteria were identified in low percentages: *Oceanospirillaceae* with the species *Marinomonas arenicola* and *Marinomonas pontica*, which are halophilic and widely distributed in marine environments and can be found in raw milk [65], and *Pasteurellaceae* with the genus *Mannhemia*, which is an ovine mastitis pathogen [66], present in one sample.

Finally, the species *Enhydrobacter aerosaccus*, which belongs to the phylum Proteobacteria but not to a specific family (characterized as *Incertae sedis*) was identified in all the samples (0.01–2.48%). *Enhydrobacter aerosaccus* is an aquatic Gram-negative facultative anaerobic and gas-vacuolated rod [67] that has been commonly found in sheep and goat milk [61].

The phylum Bacteroidetes was the third most abundant, represented by four genera (*Chryseobacterium*, *Flavobacterium*, *Prevotella*, *Elizabethkingia*). *Chryseobacterium* occur frequently in dairy products and are considered psychrotrophic spoilage bacteria [50]. The most common species present in six samples was *Chryseobacterium haifense*, which is an aerobic Gram-negative bacterium isolated for the first time from raw milk in Israel [68]. Other species present were *C. carnipullorum* and *C. ureilyticum*. *Flavobacterium*, which is also a psychrotolerant microorganism found in milk [27] but considered a previously overlooked genus in the cheese microbiome [8], was detected in three samples, with *F. hercynium* being the only species identified. *Elizabethkingia*, which is an environmental contaminant, has been also detected in the rare biosphere of cheese in other studies [58], and *Prevotella* is an anaerobic bacterium identified for the first time in Irish artisanal cheese in 2012 [69].

## 5. Conclusions

The statistical analysis of the NGS data of the PDO feta cheese commercial samples revealed a heterogenic community of 59 bacterial species and showed that microbial fingerprinting of a fermented product such as feta cheese could be feasible between different and even neighboring production areas, in this case, Epirus and Thessaly. The analysis is more thorough if the starter culture bacteria species that are found in dominant populations are excluded. However, it should also be noted that statistically significant heterogeneity was also found when the starter culture bacteria were included in the analysis. Moreover, it is confirmed that through the culture-independent NGS technique, it is possible to investigate and capture with extreme detail the microbial microbiota of feta cheese in a way that no culture-dependent methods can do. This information is basically a snapshot of a bigger and much more complex picture of the microbial communities of fermented dairy products, still useful since the various microorganisms which are either endogenously present (e.g., in milk) or exogenously retrieved from contaminated processing environments play an important role in the final organoleptic properties and the overall quality of cheese.

## Figures and Tables

**Table 1:** Species of nonstarter lactic acid bacteria (NSLAB) identified in the feta cheese samples.

Family	Nonstarter Lactic Acid Bacteria Identified	No. of Samples Detected	% Relative Abundance
Carnobacteriaceae	*Carnobacterium divergens*	1	0.02
	*Carnobacterium maltaromaticum*	1	0.03
Lactobacillaceae	*Lacticaseibacillus casei* (former *Lactobacillus casei*)	1	0.32
	*Lacticaseibacillus paracasei* (*Lactobacillus paracasei*)	6	0.01–2.48
	*Lacticaseibacillus zeae* (former *Lactobacillus zeae*)	4	0.01–0.38
	*Lactobacillus helveticus*	1	0.04
	*Latilactobacillus curvatus* (former *Lactobacillus curvatus*)	2	0.01–0.02
	*Latilactobacillus graminis* (former *Lactobacillus graminis*)	1	0.02
	*Latilactobacillus sakei* (former *Lactobacillus sakei*)	1	0.03
	*Leuconostoc lactis*	1	0.02
	*Leuconostoc* *pseudomesenteroides*	1	0.02
	*Levilactobacillus brevis* (former *Lactobacillus brevis*)	4	0.01–0.07
	*Limosilactobacillus fermentum* (former *Lactobacillus fermentum*)	1	0.01
	*Pediococcus parvulus*	2	0.01–0.057
Streptococcaceae	*Lactococcus piscium*	6	0.03–0.72
	*Lactococcus raffinolactis*	8	0.05–0.67
	*Streptococcus gallolyticus*	1	0.01
	*Streptococcus parauberis*	1	0.05
	*Streptococcus salivarius*	1	0.01
	*Streptococcus uberis*	5	0.09–0.16
	*Streptococcus vestibularis*	2	0.01–0.04

## Data Availability

Not applicable.
